# Fascia iliaca block in the emergency department for hip fracture: a randomized, controlled, double-blind trial

**DOI:** 10.1186/s12877-019-1193-0

**Published:** 2019-07-01

**Authors:** Mathieu Pasquier, Patrick Taffé, Olivier Hugli, Olivier Borens, Kyle Robert Kirkham, Eric Albrecht

**Affiliations:** 1 Department of Emergency Medicine, Lausanne University Hospital, Lausanne, Switzerland; 2 Statistician, Institute of Social and Preventive Medicine (IUMSP), Lausanne University Hospital, Lausanne, Switzerland; 3 Department of Orthopaedic surgery and Traumatology, Lausanne University Hospital, Lausanne, Switzerland; 4 Department of Anaesthesia, Toronto Western Hospital, Toronto, Canada; 5 Department of Anaesthesia, Lausanne University Hospital, Rue du Bugnon 46, BH 05.311, 1011 Lausanne, Switzerland

**Keywords:** Analgesia, Hip fractures, Lumbosacral plexus

## Abstract

**Background:**

Hip fracture causes moderate to severe pain and while fascia iliaca block has been reported to provide analgesic benefit, most previous trials were unblinded, with subsequent high risks of performance, selection and detection biases. In this randomized, control double-blind trial, we tested the hypothesis that a fascia iliaca block provides effective analgesia for patients suffering from hip fracture.

**Methods:**

Thirty ASA I-III hip fracture patients over 70 years old, who received prehospital morphine, were randomized to receive either a fascia iliaca block using 30 ml of bupivacaine 0.5% with epinephrine 1:200,000 or a sham injection with normal saline. The fascia iliaca block was administered by emergency medicine physicians trained to perform an anatomic landmark-based technique. The primary outcome was the comparison between groups of the longitudinal pain score profiles at rest over the first 45 min following the procedure (numeric rating scale, 0–10). Secondary outcomes included the longitudinal pain score profiles on movement and the comparison over 4 h, 8 h, 12 h, and 24 h after the procedure, along with cumulative intravenous morphine consumption at 24 h.

**Results:**

At baseline, the fascia iliaca group had a lower mean pain score than the sham injection group, both at rest (difference = − 0.9, 95%CI [− 2.4, 0.5]) and on movement (difference = − 0.9, 95%CI [− 2.7; 0.9]). These differences remained 45 min after the procedure and the two longitudinal pain score profiles were parallel both for patients at rest and on movement (test of parallelism for patients at rest *p* = 0.53 and on movement *p* = 0.45). The same parallel change in pain scores over time was observed over 24 h of follow-up (test of parallelism for patients at rest *p* = 0.82 and on movement *p* = 0.12). These results were confirmed after adjustment for gender, ASA score, and cumulative sums of intravenous morphine received pre-procedure and during-follow-up. In addition, there was no difference between the two groups in total cumulative intravenous morphine consumption at 24 h.

**Conclusion:**

Fascia iliaca block following anatomic landmarks may not provide supplementary analgesia for patients suffering from hip fracture, when low pain scores are reported after prehospital morphine. Additional larger trials will help reach definitive conclusion.

**Trial registration number:**

Clinicaltrials.gov – NCT02433548. The study was registered retrospectively.

## Background

Hip fracture is very common in the elderly, with an incidence of 4.6 per 1000 adults over 50 years old [1], and accounts for as many as 8.9 per 1000 emergency department visits [2]. Hip fracture causes moderate to severe pain, but pain management has often been reported as insufficient [2]. Under-treatment of acute pain in emergency medicine may affect over 40% of patients in both prehospital and in-hospital settings, and extends to the pain management of hip fracture [3–5]. Inadequate pain management results from several factors, including underestimation of pain intensity by healthcare providers, insufficient administration of analgesics due to the fear of side-effects or under-estimation of the doses required to treat moderate to severe pain [6]. Unfortunately, unrelieved pain increases the risk of delirium among patients suffering from hip fracture by a factor of nine [7], and has prompted investigation of alternative pain treatments, such as regional anaesthetic techniques [8].

The hip joint is innervated by the femoral and obturator nerves anteriorly, and the sciatic nerve posteriorly [9]. The fascia iliaca block (FIB) was first described in 1989 and consists of injecting local anaesthetics below the fascia iliaca and lateral to the femoral vessels, purportedly resulting in anaesthesia of the lateral cutaneous, femoral and obturator nerves [10]. Since then, FIB has been reported to provide adequate analgesia in hip fracture case series [11, 12]. It has also been stated that FIB can be easily performed by non-anaesthesiologists, given the very low risk of complications [11, 13]. Despite these reports, evidence suggests that less than one third of physicians in emergency departments perform this regional block [14], due mainly to a lack of supporting evidence [15]. Although a recent meta-analysis concluded that this technique provides analgesic benefit [16], the conclusion is limited by the absence of blinding in 6 out of 8 included articles, with subsequent high risks of performance, selection and detection biases [8, 17–21]. The two remaining articles arguably overstated their conclusions [22, 23], as a significant difference was present at only a single time interval. The magnitude of analgesic efficacy of FIB for patients suffering from hip fracture therefore remains unanswered.

In this randomized, controlled double-blind trial, we tested the hypothesis that landmark-based FIB for hip fracture, performed by emergency medicine physicians, provides effective analgesia.

## Methods

Ethical approval was granted by the ethics committee of the Lausanne University Hospital, Switzerland (Commission cantonale d’éthique de la recherche sur l’être humain, study ID number: 318/14) on October 10, 2014, and the trial was registered on Clinicaltrials.gov (NCT02433548) on October 20, 2014. The CONSORT statement was followed in reporting this trial [24]. The study took place in the Emergency department of the Lausanne University Hospital between November 7, 2014 and June 2, 2016. Study participation was proposed to patients over 70 years old who were admitted for to the emergency department with fractured hip. Exclusion criteria were bleeding disorder or presence of anticoagulation, periprosthetic fracture, a known polyneuropathy, body weight below 40 kg, chronic pain condition, patients undergoing chemotherapy, infection at the site of injection, allergy to local anaesthetics and cognitive disorder.

After written informed consent was obtained, subjects were randomly allocated to either the experimental group (FIB group) or the control group (sham injection group) according to a computer-generated list of random numbers (www.randomization.com, seed 20,388). Assignments were concealed in a sealed opaque envelope. An emergency medicine physician, who was not responsible for the patient’s care, prepared the study drugs in syringes and performed the block procedure. A blinded study nurse collected the data. The physicians and nurses responsible for the patient’s care in the emergency department and on the ward were all blinded to the group allocation.

All procedures were performed in the emergency department, by emergency medicine physicians. Electrocardiogram, pulse oximetry, and blood pressure monitors were routinely applied. Oxygen was provided and peripheral intravenous access was established. Patients in the FIB group received an injection of 30 ml bupivacaine 0.5% with epinephrine 1:200,000, following an anatomical landmark-based technique previously described [10, 25]. Briefly, the site of injection was identified and marked 1 cm below the junction of the lateral and middle thirds of a line between the anterior superior iliac spine and the pubic tubercle. After sterilization with a solution of chlorhexidine 2% in isopropyl alcohol 70%, a short-bevel needle (Plexifix® 50 mm, 24 G, BBraun medical AG, Melsungen, Germany) was inserted at a right angle to the skin until 2 losses of resistance were felt, corresponding to the fascia lata and fascia iliaca piercing, respectively. The entire volume was then injected in 5 ml-increments. Patients assigned to the control group received a 5 ml-subcutaneous injection of normal saline in the same location after sterilization of the skin. All procedures were performed by physicians who had experience with the anatomical landmark-based technique and who were not further involved in the study.

After the procedure, patients received acetaminophen 1000 mg every 6 h. Persistent pain (numeric rating scale [NRS] > 3) was treated with intravenous morphine as needed. After transfer to the orthopaedic ward, patients were prescribed acetaminophen 1000 mg every 6 h and subcutaneous morphine 0.1 mg.kg^− 1^ every 6 h as needed.

We originally planned to compare the two mean pain scores (fascia iliaca block versus sham injection groups) at 45 min. However, given that, despite randomisation, the two groups had different mean baseline scores, we elected to evaluate the longitudinal pain trajectories and tested for parallelism instead. Therefore, the primary outcome was the comparison between groups of the longitudinal pain score profiles at rest over the first 45 min following the procedure. Pain intensity was assessed with an 11-point numeric rating scale (NRS), ranging from 0 (no pain) to 10 (worst imaginable pain). Secondary pain-related outcomes included comparison of the longitudinal pain score profiles on movement over the first 45 min following the procedure; longitudinal pain score profiles at rest and during movement (standardized gentle 15°-elevation of the leg) 4 h, 8 h, 12 h and 24 h after the injection (NRS, 0–10); and cumulative intravenous morphine consumption at 24 h after the injection (mg). Other outcomes were length of stay, and mortality at 3 months. The between group difference in NRS score over time was analysed as a post hoc addition to the protocol in order to explore the potential impact of baseline pain score differences between groups.

Based on published data, mean NRS pain score at rest when patients are untreated was anticipated to be 8/10 with a variance of 2 [18]. We determined a clinically meaningful decrease in pain score at rest to be 2 points after a fascia iliaca block. As a result, we calculated that a minimum of 10 patients per group was necessary to detect a difference between groups, with a power of 90% and an alpha error of 0.05, using a one-sided test. Allowing for a 50% patient drop-out rate due to protocol violation or consent withdrawal, we planned to enrol a total of 30 patients. Continuous and non-continuous data are summarized as medians with 25th–75th interquartile ranges (IQR), means and standard deviations or absolute numbers, when appropriate. Baseline characteristics of the two groups were compared using the Wilcoxon-Mann-Whitney rank sum test for continuous variables (age, pre-intervention drug dose received) and by the Pearson’s chi-squared test for discrete variables (gender, ASA score, etc.). The NRS over time was analysed using linear mixed models with a random effect to account for the correlation induced by the individual differences [26]. The analyses were adjusted for gender, ASA score, and pre-procedure intravenous morphine consumption. The between group difference in NRS was assessed over time for different follow-up periods using the Wald test. The interaction between gender and ASA category was also tested. The best dose-response functional forms for the pre-intervention cumulative intravenous morphine consumption were assessed by the method of fractional polynomials and the goodness of fit by residual analysis [27]. Significance was considered at *p* < 0.05. Statistical analyses were performed using the Stata 14.2 statistical package (Stata Corporation, College Station, Texas).

## Results

**Fig. 1:**
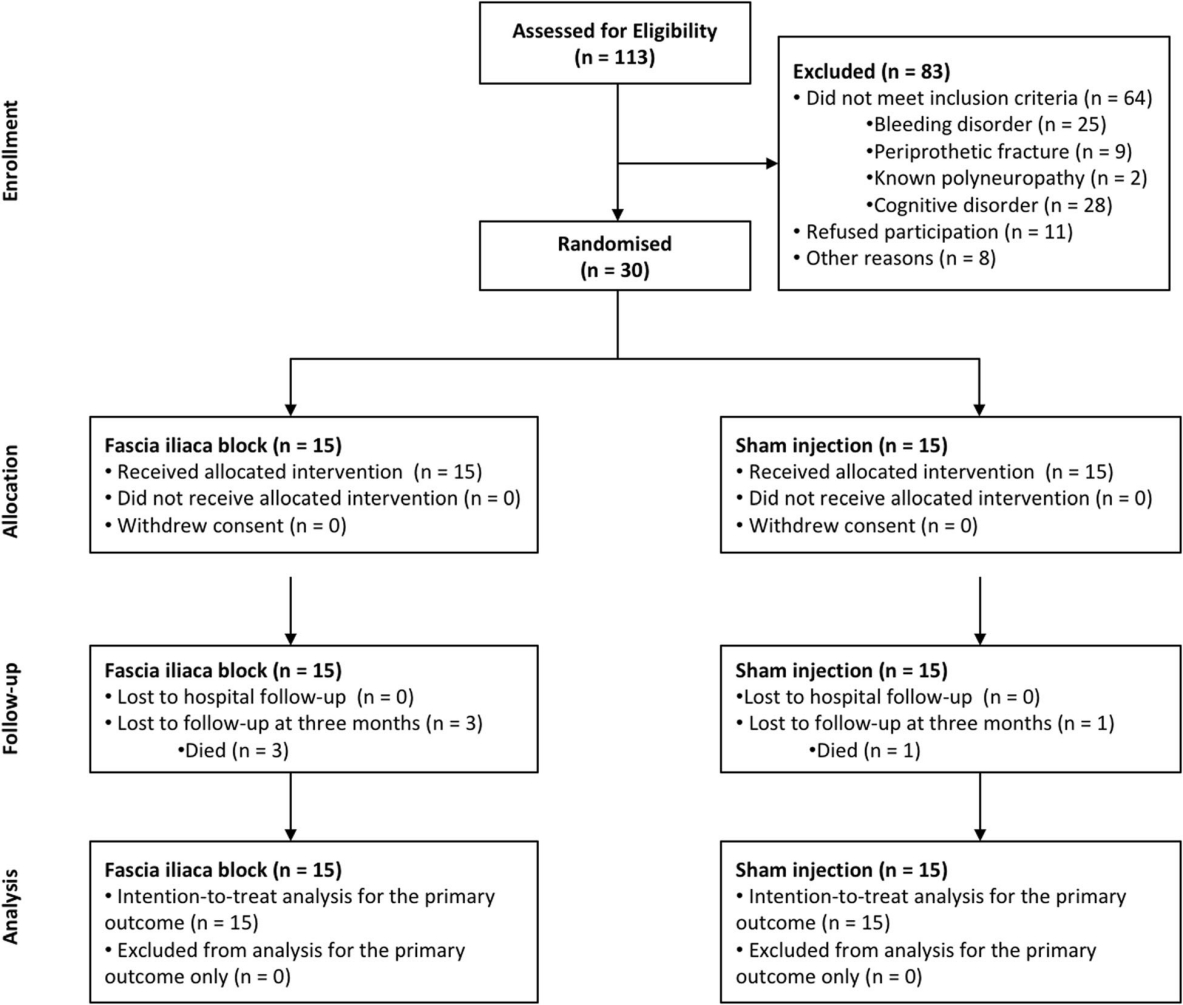
Flow of patients through the trial

Thirty patients were recruited, and all completed the follow-up for the primary outcome. Figure 1 presents the flow of patients through the trial, and Table 1 describes the patient characteristics.

**Table 1:** **Table 1**

	Fascia iliaca block group	Sham injection group
Gender (male / female)	3 / 12	6 / 9
Age (years)	78 (73–90)	83 (73–90)
Height (cm)	165 (162–173)	170 (160–180)
Weight (kg)	65 (58–80)	63 (50–74)
ASA (I / II / III)	4 / 11 / 0	2 / 12 / 1
Pre-procedure analgesic data		
Pain at rest (NRS, 0–10)	2 (1–4)	2 (2–5)
Pain on movement (NRS, 0–10)	7 (5–9)	8 (6–10)
Intravenous morphine consumption (mg)	7 (2–10)	4 (0–14)

*ASA* American Society of Anaesthesiologists, *NRS* Numeric Rating Scale

**Fig. 2:**
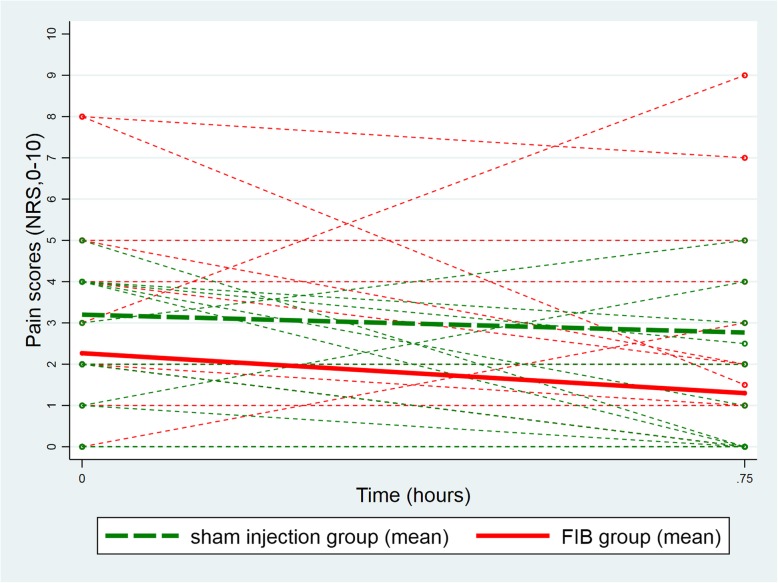
Individual evolution of pain scores at rest 45 min after the procedure

**Fig. 3:**
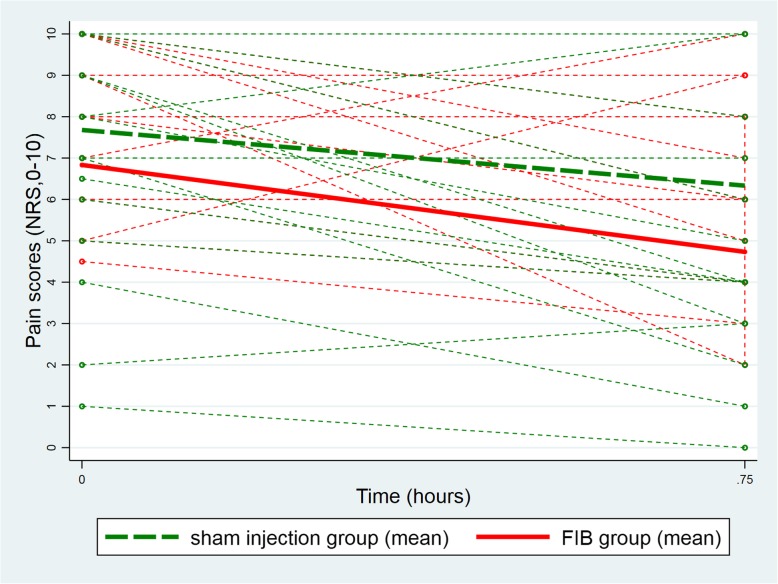
Individual evolution of pain scores on movement 45 min after the procedure

**Fig. 4:**
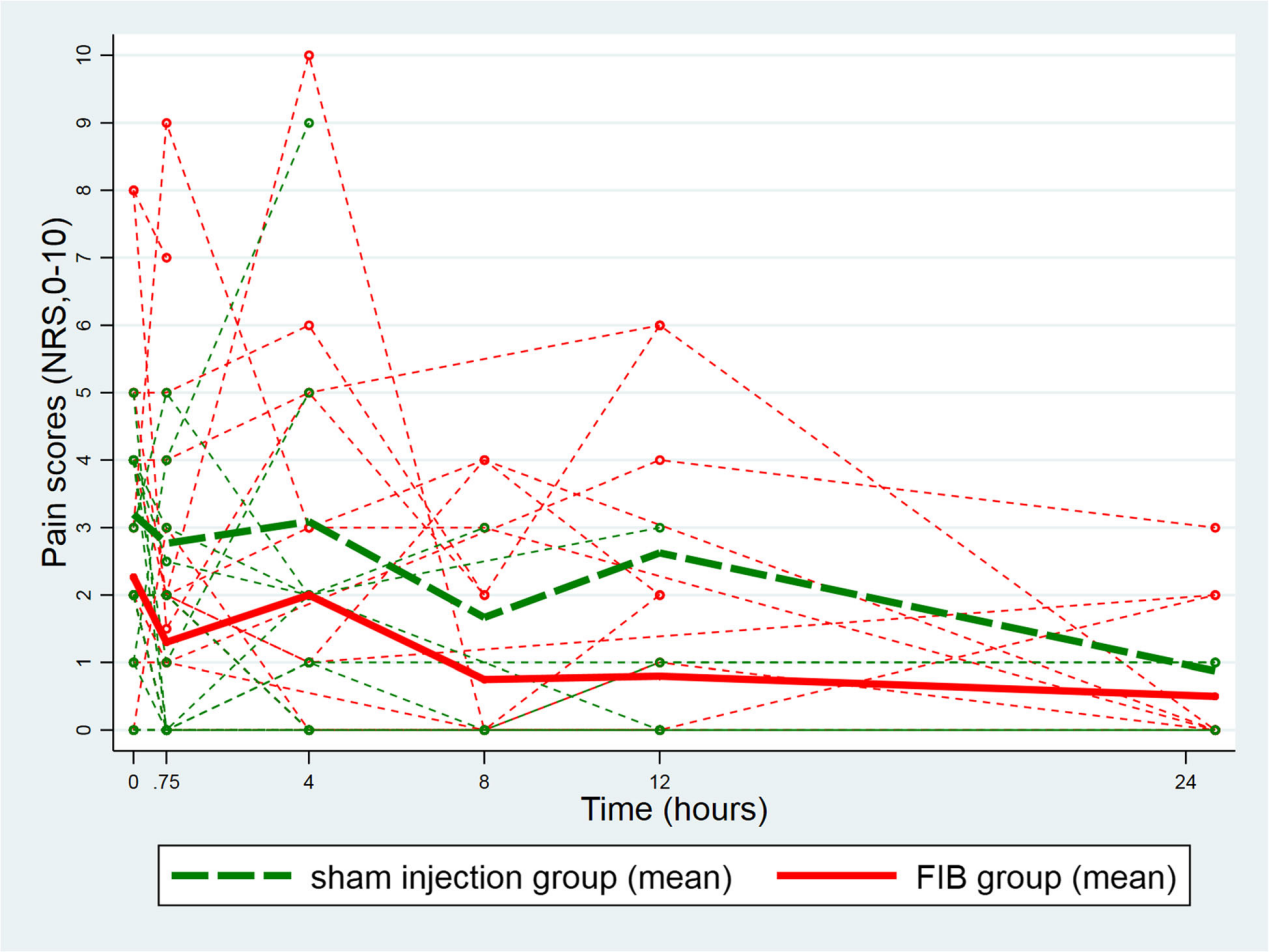
Longitudinal evolution of pain scores at rest between groups. FIB, fascia iliaca block; NRS, Numeric Rating Scale

**Fig. 5:**
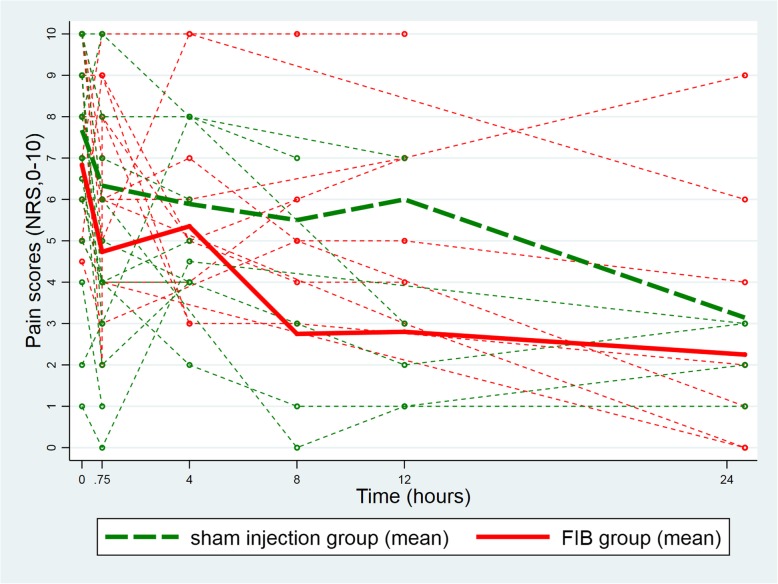
Longitudinal evolution of pain scores on movement between groups. FIB, fascia iliaca block; NRS, Numeric Rating Scale

At baseline (before the injection) the mean pain score at rest was lower in the FIB group (FIB group: 2.3, 95%CI [1.2, 3.3]; Sham injection group: 3.2, 95%CI [2.2, 4.2]; difference: -0.9, 95%CI [− 2.4, 0.5]). The difference between groups remained unchanged 45 min after the injection (FIB group: 1.3, 95%CI [0.3, 2.3]; Sham injection group: 2.8, 95%CI [1.7, 3.8]; difference: -1.5, 95%CI [− 2.9, 0.0]; test of equality of the two differences (i.e. parallelism): *p* = 0.53, Fig. 2). Similarly, the between group difference in mean pain scores on movement was also unchanged from baseline to 45 min after the procedure (baseline difference: -0.9, 95%CI [− 2.7; 0.9]; difference at 45 min: -1.6, 95%CI [− 3.4; 0.2]; test of equality of the two differences (i.e. parallelism): *p* = 0.45; Fig. 3). This equality of the two differences (i.e. the parallelism) persisted after adjustment for gender, ASA score, and pre-procedure intravenous morphine consumption (*p* = 0.50 for the test of equality of the two differences (i.e. parallelism) at rest and p = 0.45 on movement). Likewise, analyses over the periods of 4 h, 8 h, 12 h and 24 h after the procedure showed parallelism in the longitudinal pain scores at rest (*p* = 0.82; Fig. 4) and on movement (*p* = 0.12; Fig. 5), after adjustment for the same co-variables (*p* = 0.80 and *p* = 0.11, respectively).

Over the 24-h follow-up period, cumulative intravenous morphine consumption was similar between groups (FIB group: 7 mg, IQR [2; 15]; Sham injection group: 8 mg, IQR [6; 12]; *p* = 0.63), despite an apparent higher pre-procedure dose for the FIB group (FIB group: 7 mg, IQR [2; 10]; Sham injection group: 4 mg, IQR [0; 14]; *p* = 0.57), and an apparent lower cumulative dose during-follow-up (FIB group: 0 mg, IQR [0; 7.5], Sham injection group: 2 mg, IQR [0; 7]; *p* = 0.71).

Finally, the fascia iliaca block did not impact on the length of stay (FIB group: 8 days, IQR [7; 11]; Sham injection group: 9 days, IQR [7; 12]; *p* = 0.60). Three patients in the FIB group, and one in the sham injection group died at 3 months (*p* = 0.28).

## Discussion

This randomized-controlled trial suggests that anatomic landmark-based FIB does not provide additional analgesia benefit for patients in the emergency department suffering from hip fracture. While previous double-blinded investigations concluded that FIB reduces pain scores at 15 min [23] and 3 h after the procedure [22], when compared to non-steroidal inflammatory drugs or intramuscular morphine, respectively, we were unable to reproduce these results. Several factors may explain the differences seen in the present trial.

First, the reduction in pain scores in these two reports was not consistent through the timeline, as authors found a difference only at a single time interval and only for one modality of pain score [22, 23]. Second, and more importantly, we did not exclude patients who received prehospital opioids and therefore the baseline pain scores of our population were found to be much lower than those reported in the literature. Indeed, in our setting, the median pre-procedure pain score at rest was 2 (mean 3.2), while other authors have described a median of 8 [22, 23]. Of note, despite randomisation, patients in the FIB group reported a lower baseline pain score than those in the Sham injection group both at rest and on movement. This difference was explained by a higher pre-procedure morphine consumption in the FIB group. As a result, in the multivariable analyses we adjusted for both pre-procedure and post-intervention morphine consumption, in addition to ASA group and gender. Both adjusted and unadjusted analyses confirm the parallel longitudinal pain profile of the two groups, reinforcing the conclusion that FIB may not offer a significant supplementary analgesic benefit when patients report low pain scores after receiving prehospital morphine. We therefore believe that our methodology echoes the daily practice of emergency departments and reflects effectiveness of the intervention in the real-world environment.

There are several limitations associated with the present study. First, we did not assess the sensory onset of our blocks. This was omitted specifically to help maintain blinding of the group allocations. We were therefore unable to quantify a block success rate. However, all blocks were performed by emergency medicine physicians who routinely used the described procedure in daily clinical practice. Further, we recruited a total of 30 patients for the comparison of two independent means in line with our sample size calculation. However, as explained in the method section, despite randomisation, the mean baseline scores were significantly different between groups. We therefore decided to evaluate the longitudinal pain score trajectories and test for parallelism rather than a simple comparison of means. This approach, caries greater statistical power than the comparison of two means (as it is based on longitudinal data). Nevertheless, the original sample size calculation did not factor in this design and the risk of a type II error cannot be excluded. Although we adjusted for baseline characteristics such as gender, ASA score and pre-procedure intravenous morphine consumption, there may have been other unmeasured baseline characteristics, which should have been adjusted for. Our data should therefore be considered as preliminary and we suggest further research would add additional value. Finally, the choice of an anatomic landmark-based approach makes extrapolation of the study results to ultrasound-guided techniques uncertain. There is some evidence that incorporation of ultrasound may have a positive impact on the sensory blockade territory [28] and therefore may produce a different clinical outcome than our results. Future investigation into the analgesic efficacy of ultrasound-guided block, especially in light of new approaches of the lumbar plexus [29] would be beneficial.

## Conclusions

In conclusion, fascia iliaca block following anatomic landmarks may not provide supplementary analgesia for patients suffering from hip fracture, when low pain scores are reported after prehospital morphine. Additional larger trials will help reach more definitive conclusions.

## Data Availability

The datasets used and/or analysed during the current study are available from the corresponding author on reasonable request.

## Abbreviations

95%CI: 95% confidence interval
ASA: American Society of Anesthesiologists
FIB: Fascia iliaca block
h: hour
IQR: Interquartile ranges
NRS: Numeric Rating Scale
